# Targeting neurotransmitter receptors with nanoparticles *in vivo* allows single-molecule tracking in acute brain slices

**DOI:** 10.1038/ncomms10947

**Published:** 2016-03-14

**Authors:** Juan A. Varela, Julien P. Dupuis, Laetitia Etchepare, Agnès Espana, Laurent Cognet, Laurent Groc

**Affiliations:** 1 University of Bordeaux, Interdisciplinary Institute for Neuroscience, UMR 5297, Bordeaux F-33000, France; 2 CNRS, IINS UMR 5297, Bordeaux F-33000, France; 3 University of Bordeaux, LP2 NUMR 5298, Talence F-33405, France; 4 Institut d'Optique & CNRS, LP2NUMR 5298, Talence F-33405, France

## Abstract

Single-molecule imaging has changed the way we understand many biological mechanisms, particularly in neurobiology, by shedding light on intricate molecular events down to the nanoscale. However, current single-molecule studies in neuroscience have been limited to cultured neurons or organotypic slices, leaving as an open question the existence of fast receptor diffusion in intact brain tissue. Here, for the first time, we targeted dopamine receptors *in vivo* with functionalized quantum dots and were able to perform single-molecule tracking in acute rat brain slices. We propose a novel delocalized and non-inflammatory way of delivering nanoparticles (NPs) *in vivo* to the brain, which allowed us to label and track genetically engineered surface dopamine receptors in neocortical neurons, revealing inherent behaviour and receptor activity regulations. We thus propose a NP-based platform for single-molecule studies in the living brain, opening new avenues of research in physiological and pathological animal models.

## Abstract (web summary)

Single quantum dot tracking is an established technique used in cultured neurons and organotypic brain slices. Here, the authors demonstrate *in vivo* targeting of dopamine receptors via ventricular injection of functionalized quantum dots and track single nanoparticle-receptors in acute rat brain slices.

## Graphical abstract

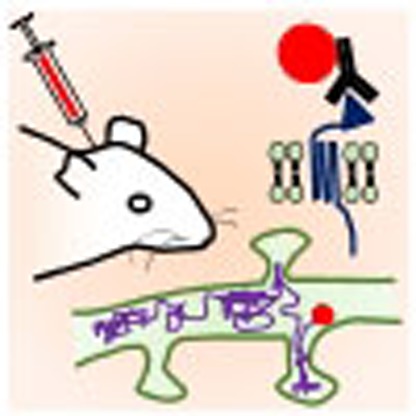

The development of new nanoprobes and imaging techniques has deeply impacted the neuroscience community over the past few years. Functionalized nanoparticles (NPs) have made possible the tracking of individual molecules in living cells, drastically changing the way we understood synaptic communication. In particular, neurotransmitter receptors have been successfully labelled with functionalized quantum dots (QD) and tracked diffusing along neurons, revealing new synaptic regulation mechanisms. Thanks to single-molecule tracking techniques, new properties of excitatory glutamate AMPA^1^,^2^,^3^ and NMDA^4^,^5^,^6^, inhibitory glycin^7^ and GABA^8^ receptors and more recently the modulatory dopamine receptors^9^,^10^ have been characterized opening up new targets for therapy. Undoubtedly, single-molecule tracking imaging approaches shed new and unexpected light on the molecular regulation of brain cell communication^11^,^12^. This approach has the advantage to identify the molecular behaviour of receptor sub-populations, even minority ones, while retrieving molecule localizations with sub-wavelength precision. In addition, the use of nanometre-sized particles has even made possible to track target molecules within confined cellular compartments^13^,^14^. However, a clear limitation of the single NP tracking approach has been the need to use cultured neuronal systems, and not intact thick brain tissue.

Recently, single-molecule tracking in neurons using NPs has been extended to cultured organotypic slices, which provide the great advantage of an easy and direct access to superficial cells^15^. Although cultured neurons and organotypic slice cultures are useful systems to investigate some neural mechanisms, they unequivocally differ in many aspects from cell networks in intact brain preparations. For instance, the architecture of the cellular assemblies is strongly altered, causing changes in the extracellular environment and intercellular communication. Extension of single-molecule tracking techniques to thick acute brain slices has thus been a major challenge that has bogged down our understanding of nanoscale dynamic organization of neurotransmitter receptors. Apart from technical difficulties regarding the imaging of single nano-objects in high background noise environments because of light scattering, absorption and tissue auto-fluorescence, targeting *in vivo* NP complexes into the brain without strong activation of the immune defense has long been an obstacle for single-particle tracking in tissue and drug delivery. Currently, NPs are delivered to the brain either through direct injection into the tissue or intravenous injection^16^,^17^,^18^. However, the direct injection produces locally a high concentration of NP that induces inflammation and activation of microglia, leading to engulfed NP. The intravenous injection of NP limits the brain delivery since only a tiny percentage is expected to cross the blood–brain barrier and reach the nervous tissue.

Here, we explored an alternative strategy that consists of injecting NP into the cerebrospinal fluid knowing that the choroid plexus epithelium is highly permeable. This delivery strategy and optimized imaging microscopy allowed us to tackle this imaging challenge and to track a surface neurotransmitter receptor at the single NP level. We concentrated our efforts on the dopamine receptor since the dopaminergic signalling in the mammalian central nervous system contributes to major functions including locomotion, novelty detection and long-term memory formation^19^,^20^. As a consequence, dysregulations of the dopaminergic system are associated with alterations in synaptic function and plasticity as well as severe neurological and psychiatric conditions such as Parkinson's disease or schizophrenia. Interestingly, new aspects of the dopamine modulation of synaptic plasticity have been recently linked to altered diffusion of surface dopamine receptors, unveiled by fluorescence recovery after photobleaching and single NP tracking approaches^10^,^21^,^22^,^23^. Single-molecule tracking of dopamine receptors in acute brain slices will offer new avenues of research and potentially new therapeutical strategies.

## Results

### NP brain-tissue delivery without major inflammatory reaction

**Figure 1:**
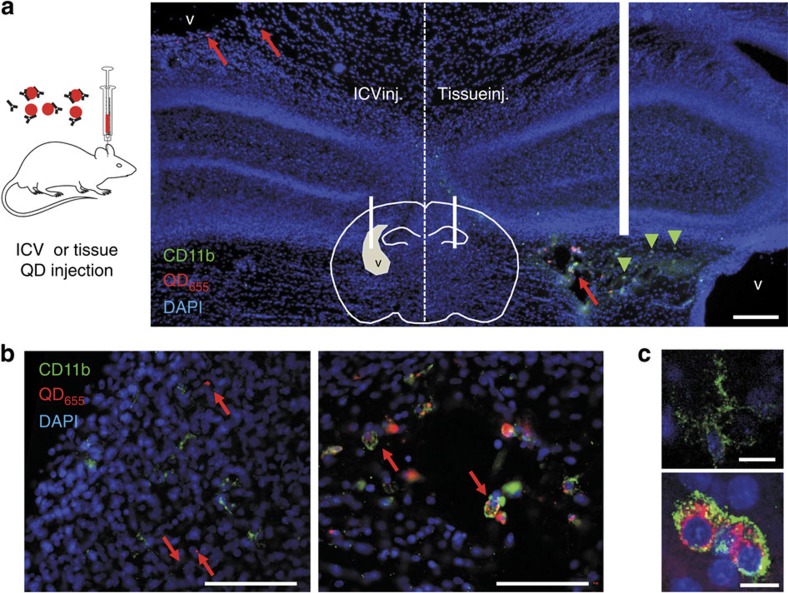
Comparison between intra-ventricular injection and local brain-tissue injection of QD. (**a**) Rats were injected with functionalized QD (shown in red) inside a lateral ventricle in one hemisphere and directly in brain tissue in the opposite hemisphere. Pups were perfused with PFA 3 h after injection, and coronal cuts were immunostained with CD11b antibodies (shown in green). Nuclei were stained with DAPI (shown in blue); scale bar, 200 μm. (**b**) Magnified images of both hemispheres (from regions marked with red arrows) are shown below, corresponding to a region close to the lateral ventricle (left) and a region close to the brain-tissue injection point (right), where activated microglia are detected with large amount of phagocyted QD (scale bars, 100 μm). (**c**) The morphology of microglia with high concentration of engulfed QD changes dramatically due to cell activation (top: non-activated; bottom: activated), also increasing the levels of CD11b expressed by microglia (scale bars, 10 μm). ICV, intra-cerebroventricular.

We chose far-red emitting QD (with an emission maximum at 655 nm) to facilitate their detection deep in living brain slice, taking advantage of the relatively low tissue auto-fluorescence, light absorption and scattering of tissues at these wavelengths. QD functionalized with anti-rabbit F(ab')_2_ fragments were conjugated with polyclonal antibodies directed against GFP 1 h before brain injection. To deliver QD to the brain tissue via the cerebrospinal fluid, intra-ventricular injections of QD were performed in newborn Sprague Dawley rats (1–4 days old). To compare the impact of a local brain injection with an intra-ventricular injection, we performed both types of injections in the same animal (one in each hemisphere). Three hours after QD administration a massive activation of microglia was observed in the injected tissue, as shown by high CD11b staining and striking changes of microglia morphology (Fig. 1a–c). Expectedly, those microglial cells contained a high amount of internalized QD (Fig. 1a,b). Consistently, almost no QD were observed outside microglia. In contrast, the contralateral hemisphere (that is, ventricular injection) shows microglia with classical non-activated state and morphology, and most QD were scattered in the tissue without specific co-localization with microglial cell markers. These data indicate that ventricular injection of QD prevents microglial activation observed after classical intra-tissue injection, allowing the dispersion and diffusion of QD within the tissue of interest.

### Brain distribution of untargeted QD after injection

**Figure 2:**
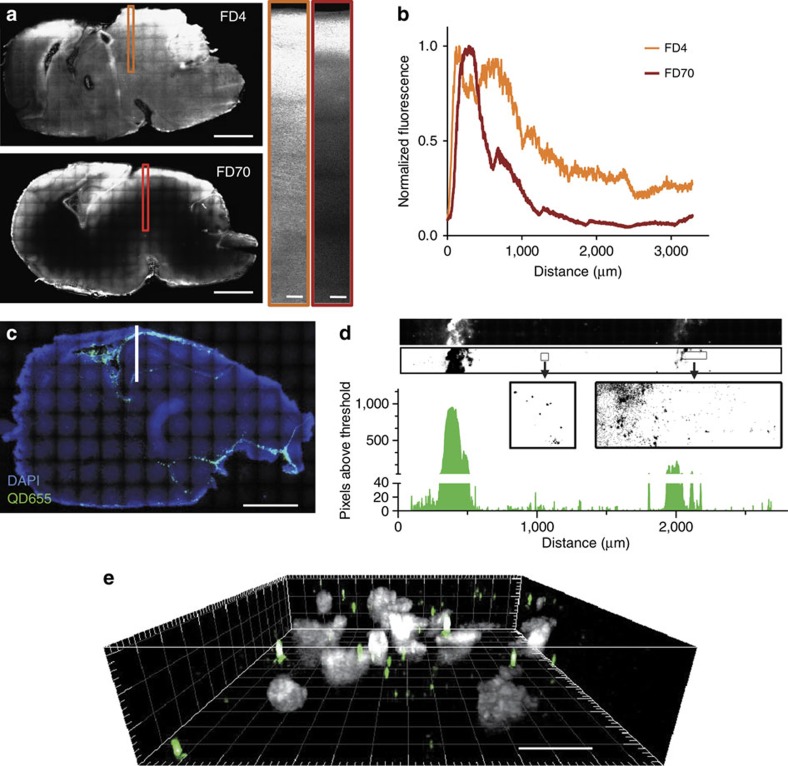
Distribution map of injected Dextran and QD in rat brain. (**a**) Intra-ventricular injections of FD4 (top) and FD70 (bottom) allow diffusion of dextran into the brain tissue (full-brain scale bars, 2 mm, magnified sections' scale bars, 100 μm). Smaller dextran (FD4) diffuses faster and further away from the ventricles than larger dextran (FD70), as can be seen in the plotted intensity profiles (**b**) corresponding to the intensity detected across the marked orange and red rectangles in the brain sections shown. (**c**) Intra-ventricular injections of QD show similar diffusion patterns to that of injected fluorescent dextran (particularly FD70). The image corresponds to a low magnification map of a 50 μm thick sagittal cut of P1 rat brain, injected with QD and killed and fixed with PFA 3 h later (scale bar, 2 mm). The map was composed stitching 120 images taken at × 10 magnification in a spinning disk confocal microscope (DAPI is shown in blue, QD shown in green). High-magnification image showing QD in the brain region marked with white line (including part of cortex, ventricle and hippocampus) is shown in **d**. The high magnification map was composed stitching 90 images taken at × 100 magnification in a spinning disk confocal microscope, subsequently thresholded and the pixels above threshold quantified, showing how QD diffuse into the brain hundreds of microns away from the cerebrospinal fluid ventricles. A section of this high-magnification image was studied in three dimensions (**e**) showing that QD (green spots) are distributed across the slice volume (DAPI in grey) and the detected QD in the brain are not dispersed as an artefact of the slicing procedure (scale bar, 10 μm).

To characterize the distribution pattern of injected QD, rats were killed 3 h after intra-ventricular injections and brains were subsequently dissected for imaging. Fluorescence images were recorded at video rate in the brain slices using a spinning disk fluorescence microscope, with three-dimensional (3D) capabilities. Smaller fluorescent nano-objects (fluorescein isothiocyanate-dextran), of average molecular weight of 4 kDa (FD4) and 70 kDa (FD70) with estimated mean diameters of 3 and 15 nm, respectively^24^, were also injected into ventricles. From sagittal brain slices, FD4 and FD70 had a stronger intensity close to ventricles (Fig. 2a,b). The fluorescence intensity decayed as the dextran diffused into the brain tissue, with an expected larger penetration depth for smaller dextran (Fig. 2b). Injected QD (Fig. 2c) were detected with a similar diffusion pattern to the one observed for FD70. At low magnification, QD appear to be rapidly dispersed in the ventricular system, as also seen in the sagittal brain slice (Fig. 2c). At high magnification, QD diffused into the brain tissue hundreds of microns away from the main ventricles (Fig. 2d). It should be mentioned that the brain ventricular network is complex and ramified, precluding a strict measurement of the distance travelled by QD away from ventricular network. Finally, to confirm that the detected QD were not dragged out from the cerebrospinal fluid during the slice preparation procedure, 3D images were performed showing evenly dispersed QD in the volume (Fig. 2e).

### Detection of single QD in acute brain slices

**Figure 3:**
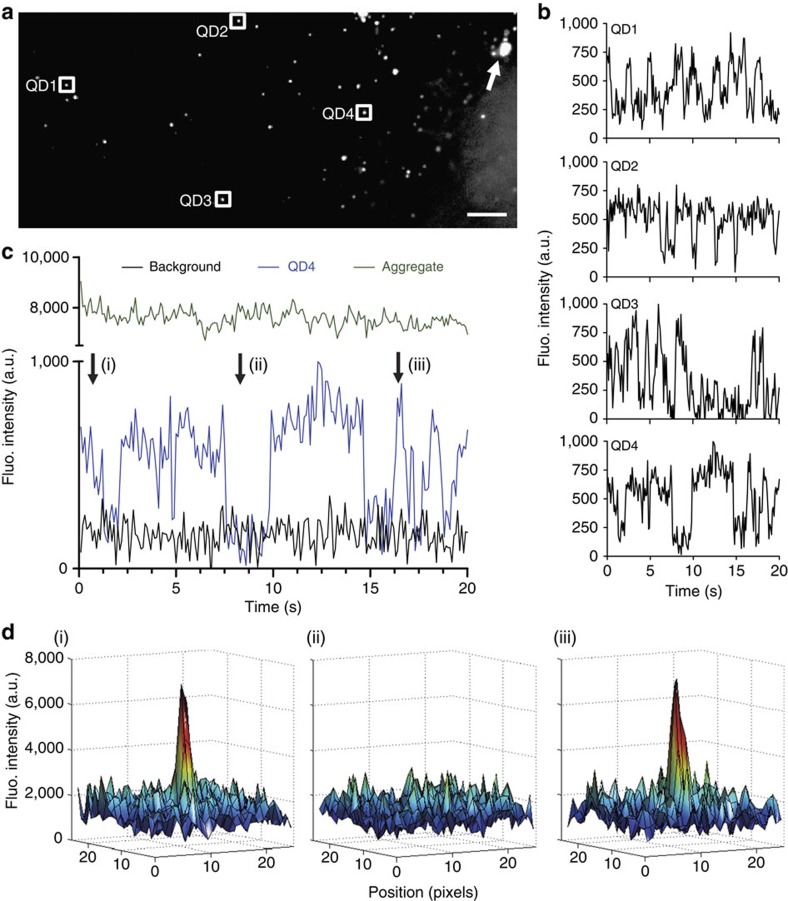
Single QD can be detected in fixed brain slices. (**a**) Spinning disk confocal image from a hippocampal region of a 50 μm thick rat brain slice injected with QD (scale bar, 15 μm). The image corresponds to the average of 200 frames. (**b**) Time behaviour of four different QD (labelled as 1–4 in **a**), showing that blinking is not synchronized and therefore, not an artefact due to stage vibrations. (**c**) Blinking behaviour of the fluorescence of a single QD (blue line), of an aggregate of QD (green line, shown with an arrow in **a**), and background fluorescence (black line). (**d**) Examples of the fluorescence intensity of QD4 at times indicated with arrows in (**c**).

**Figure 4:**
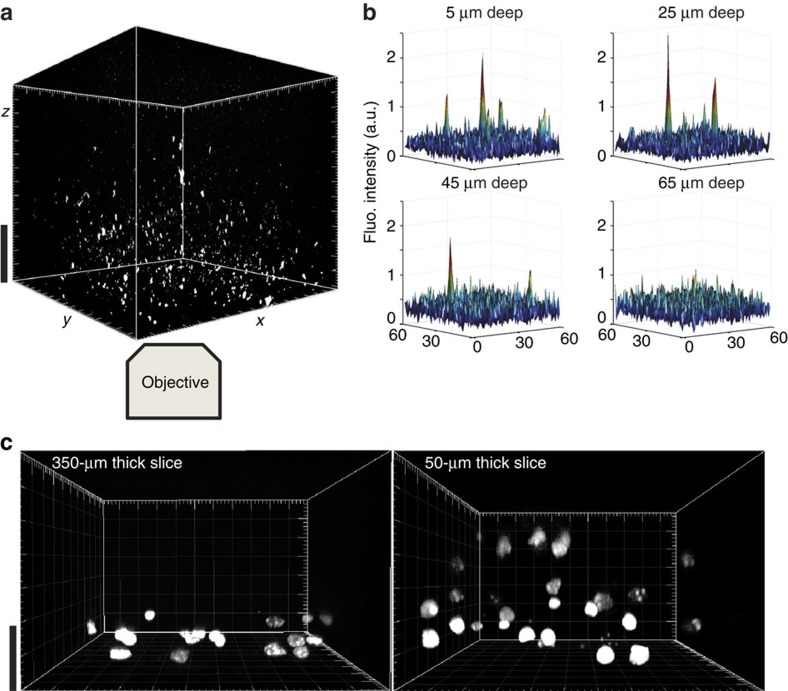
Imaging penetration depth analysis of the detection of single QD in acute brain slices. (**a**) 3D reconstruction of *z*-stack acquired with spinning disk confocal microscopy in an acute brain slice of a rat injected with QD (scale bar, 20 μm). The imaged QD can be detected in live tissue at least up to depths of 45 μm (**b**), as exemplified with surface view of the image pixel levels. Membrane permeability assays were performed with PI staining, showing a layer of damaged cells in 350 μm slices and much more damage in 50 μm thick slices (**c**) (scale bar, 20 μm).

Single QD display a good photo-resistance allowing for long recordings (tens of seconds) and displaying a characteristic blinking behaviour^14^,^25^. We took advantage of these properties to reveal that single QD could be detected in our brain-tissue preparations with the spinning disk setup operating at a rate of 10–30 frames per second (Fig. 3a–d). In particular, we checked that the blinking behaviour of different QD identified in an imaging field was not time-correlated ruling out the possibility that the observed blinking was an out-of-focus imaging because of stage vibrations (Fig. 3c). Importantly, single QD were detected in acute slices at depths up to ∼50 μm inside the slice, corresponding approximately to 3–4 pyramidal cell layers inside the slice (Fig. 4a,b). The imaging depth capability was verified in an independent assay, in which thinner acute slices (50-μm thick slices prepared with a vibratome) were used to detect single QD along the entire *z* axis of the slice (Supplementary Fig. 1). We also performed a membrane permeability assay by adding propidium iodide (PI) to the artificial cerebrospinal fluid (ACSF) while imaging brain slices. In 350-μm thick acute brain slices, 3D reconstructed image shows that only the very first layer contains some stained nuclei while deeper layers are composed of PI-negative healthy cells (Fig. 4c). Performing the same staining in 50-μm thick slices demonstrate the presence of PI-positive dead cells, scattered along the entire volume of the slice (Fig. 4c). Thus, 350-μm thick acute brain slices were exclusively used and experiments were performed at depth above 20 μm within slices.

### Characterization of QD-antibody complex

Cultured neurons expressing CFP-dopamine D1R (CFP located at the extracellular N-terminus of the receptor) and EGFP plasmids (to fill transfected neurons with a fluorescent reporter) were incubated with QD-antibody complexes at various ratio of QD and antibodies. First, we verified that the use of polyclonal antibody did not alter *per se* the diffusion of surface D1R, as suggested for other surface receptors when used at higher concentrations^26^. Comparing the surface dynamics of D1R targeted by either monoclonal or polyclonal antibodies showed equal diffusion coefficient distributions (Supplementary Fig. 2a). It should also be noted that we previously demonstrated that the bivalence of antibody at this high dilution does not impact the receptor trafficking as compared with monovalent probes^14^. We then tested the impact of various ratios of QD and antibody concentrations on receptor dynamics. For this, QD-anti-GFP complexes of different QD/antibody ratios (1:1, 1:10, 1:1,000) were added to cultured neurons expressing D1-CFP and QD trajectories were analysed. The comparison between conditions shows that only a high concentration of anti-GFP (1:1,000) slows down D1R surface dynamics, likely due to receptor crosslinking (Supplementary Fig. 2b). On the basis of this knowledge, we then used the 1:10 ratio in the rest of the study to perform our *in vitro* and *in vivo* single QD tracking.

### Single QD-D1 receptor detection in acute brain slices

**Figure 5:**
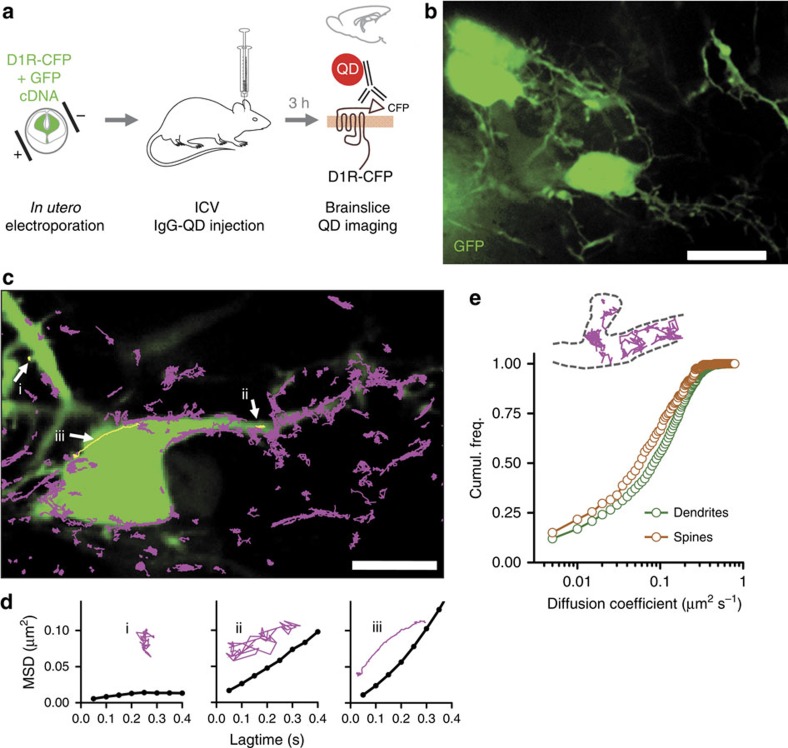
Single D1 receptor tracking in acute brain slices. (**a–b**) Rats were electroporated *in utero* with D1-CFP and EGFP constructs and electroporated pups were injected with QD to recognize the CFP epitope in transfected neurons. Acute brain slices of electroporated animals injected with functionalized QD were imaged in a spinning disk confocal microscope (**b**) (scale bar, 15 μm). (**c**) Fast two-dimensional imaging was performed over transfected neurons with coupled QD and subsequently QD were identified and trajectories reconstructed (scale bar, 10 μm). (**d**) Example trajectories with the calculated mean squared displacement. (**e**) A comparison between the diffusion of QD in spines and dendrites shows that the calculated coefficients are slower for spines (*n*=212 trajectories along spines, *n*=2,193 trajectories along dendrites).

*In utero* electroporation was performed in pregnant Sprague Dawley rats at day 16.5 of gestation, double-transfecting brain embryos with CFP-D1R (CFP located at the extracellular N-terminus of the receptor) and EGFP plasmids. Pups (1–4 days old) received an intra-cerebroventricular injection of a QD/anti-GFP antibody complex that recognizes the CFP motif of CFP-D1R (Fig. 5a)^9^,^10^. Three hours after injection, pups were killed, sagittal brain slices were prepared in ACSF and electroporated neurons were detected in the neocortex, that is, both in the hippocampus and cortex (for example, Fig. 5b). QD were detected throughout the neocortex (for example, Supplementary Movie 1) and imaged over 500 frames to retrieve individual QD trajectories. Strikingly, numerous QD were found to rapidly diffuse along GFP-positive dendrites (Fig. 5c, Supplementary Movie 2), therefore likely specifically attached to surface D1R. Their motion type was indeed different from those of single QD found to be stuck to the membrane (immobile) or rapidly diffusing in the extracellular space. However, since there is no easy way of washing away unbound QD inside living brains or slices (as achieved in cultured neurons or organotypic slices) the ‘unbound' fraction of anti-GFP-QD were not further considered. QD trajectories diffusing along GFP-positive neurons were analysed by calculating the mean squared displacement (MSD) revealing that some QD movements were confined to spines (for example, Fig. 5di), whereas others moved more freely onto the dendritic shaft (for example, Fig. 5dii). Interestingly some active transport (for example, Fig. 5diii) was also found but at very low frequency. Remarkably, we could identify D1R-QD moving from dendrites to spine heads (for example, Supplementary Movie 2), with the diffusion coefficients of QD in spines lower than those on dendrites (Fig. 5e).

**Figure 6:**
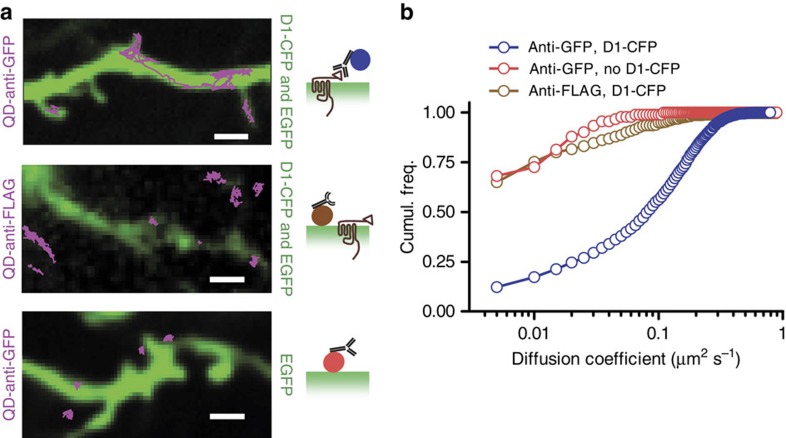
Specific and unspecific labelling. (**a**) Some QD functionalized with anti-GFP were found to diffuse over large dendritic areas, inside and outside spines of neurons transfected with D1-CFP (scale bar, 2 μm). QD functionalized with anti-Flag antibodies did not show diffusion patterns along D1-CFP transfected neurons, but mainly appeared immobile when co-localizing with transfected neurons. The same immobile pattern was observed with QD functionalized with anti-GFP over neurons that were not transfected with D1-CFP. (**b**) A quantification of these different behaviours can be seen in the cumulative distribution of diffusion coefficients.

To further test the specificity of QD targeting, we injected QD functionalized with an anti-FLAG antibody instead of the anti-GFP antibody that can bind to CFP-D1R. The imaging of single QD/anti-FLAG complexes in electroporated brains (EGFP+D1R-CFP) did not show trajectories diffusing along GFP-positive neurons (Fig. 6, Supplementary Movie 3), supporting the specificity of the trajectories observed using anti-GFP functionalization. In another series of experiments, we injected QD functionalized with an anti-GFP antibody into brains electroporated only with EGFP (no D1R-CFP). Similarly, there was no trajectory diffusing along GFP-positive (Fig. 6). The diffusion coefficient distributions of these conditions show the marked difference between ‘specific' and ‘unspecific' staining of D1R (Fig. 6). Altogether, the diversity of behaviours observed by single NP tracking in brain slices strengthen the view that neurotransmitter receptors, such as D1R, diffuse at the surface of neurons in a compartment-dependent manner, and that the high exchange rate between synaptic and extra-synaptic areas that was revealed in cultured neurons, is now confirmed in intact brain tissue and is thus more than likely a key parameter of receptor trafficking and synaptic physiology.

### Regulation of D1/5R surface dynamics in acute slice

**Figure 7:**
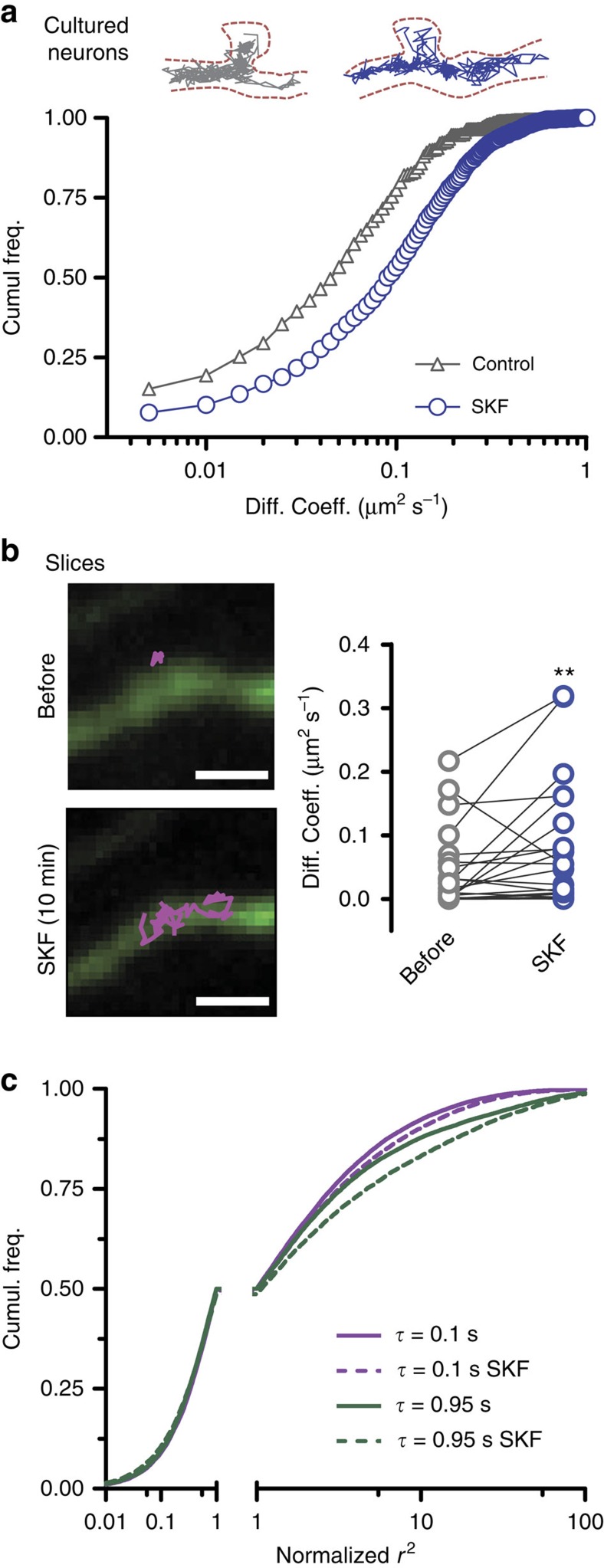
Effect of SKF-38393 on D1R diffusion. (**a**) The effect of the D1 agonist SKF-38393 (10 μM for 10 min) was tested in cultured neurons (control, *n*=1,205 trajectories; SKF-38393, *n*=1,174 trajectories; *P*<0.001 Kolmogorov–Smirnov test, showing a shift in the diffusion coefficient distribution. (**b**) The effect was also compared pairwise in acute slices based on QD that could be detected before and after treatment with SKF-38393 (as illustrated in the snapshots, scale bar, 2 μm), showing a significant increase in diffusion (*n*=24, control D=0.04±0.01 μm^2^ s^−1^, SKF-38393 D=0.07±0.02 μm^2^ s^−1^, Wilcoxon matched-pairs signed rank test *P*=0.0099). (**c**) Step distribution analysis of acute slices was performed for various lag times (example squared step distributions for lag time *τ*=0.1 s and 0.95 s) for control and SKF-38393 conditions. The squared step axes were normalized to 1 at the 0.5 cumulative distribution point in order to make a direct visual comparison of the populations (control, *n*=28,404 steps; SKF-38393, *n*=28,367 steps).

In cultured neuronal systems, the surface dopamine D1R are highly regulated by the receptor activity status and associated intracellular signalling^10^,^21^. We thus investigated the effect of the dopamine D1R and D5R agonist SKF-38393 on D1R-QD mobility in intact brain tissue. In cultured hippocampal neurons, D1R diffusion coefficient distribution was shifted towards higher values after SKF-38393 (10 min and 10 μM), corresponding to a 55% increase in the median diffusion coefficient (Fig. 7a). Remarkably, we tracked surface D1R before and after SKF-38393 exposure in acute slices and observed a 53% increase in the diffusion coefficient after activation (Fig. 7b). It is important to note that QD complexes that did not co-localize with GFP-positive neurons showed no significant change in their diffusion behaviour (Supplementary Fig. S3).

We then aimed at better characterizing the diffusion regulation observed in the acute slice preparation. One must notice that MSDs of individual neuronal membrane molecules display high variability and are subject to statistical uncertainties due to the limited length of the trajectories. This renders the comparison of diffusion constants eventually blurred by the diverse types of movements of individual receptors. We thus chose a previously developed statistical approach to analyse globally the mobility behaviours of sub-populations^27^, regardless of the trajectory length. It consists in analysing the probability distributions of the square displacements *r*^2^ (denominated ‘squared steps') performed by the molecules during a given time interval *τ* pooling together all trajectories. Different populations of mobile molecules were identified at all *τ* characterizing the evolution of squared steps for increasing time interval *τ* (Fig. 7c and Supplementary Fig. 4a,b). Displaying the step distributions at different time intervals (for example, 0.1 s and 0.95 s), it clearly appeared that application of SKF-38393 affects the faster diffusing population (large step portion of each distribution), whereas no effect was observed for the slowly mobile population (Fig. 7c). Altogether, these data provide the first direct evidence that the activation of D1R in an intact brain slice specifically modulates the receptor surface dynamics of the fast diffusing sub-population, which have often been associated with recycling and modulatory pool^10^,^28^.

## Discussion

Since the initial discovery of the lateral diffusion of neurotransmitter receptors using single-particle tracking^7^, the surface diffusion of receptors and other membrane molecules has been extensively studied in brain cell cultures, unravelling for instance its key role in excitatory and inhibitory synapse adaptations^3^,^5^,^29^,^30^. Semiconductor QD have been the preferred NP to label and track a wide variety of biomolecules diffusing along the plasma membrane^3^,^25^,^31^,^32^,^33^ of cultured glial cells^34^ and neurons^2^,^25^,^29^. While photobleaching-based approaches were commonly used to confirm the global dynamic properties of surface receptors in brain slices^3^,^5^, QD delivery issues currently limited the transfer of single-particle tracking techniques to more integrated and preserved preparations, limiting investigations of receptor diffusion in the context of native tissue and fostering intense debate in the neurobiological field on the existence and relevance of surface receptor dynamics in intact brain tissue. Recently, QD tracking of surface lipids and receptors has been nicely extended to organotypic brain slices since the preparation provides most of the advantages of cultured systems^15^. Here, we report a technical development that allows to track with high pointing accuracy the dynamics of surface tagged-receptors in intact acute brain tissue, demonstrating that dopamine receptor diffusion is finely regulated upon receptor activation.

An efficient method to deliver NP to the brain should benefit a variety of research lines in the fields of neurobiology and medicine, such as the delivery of encapsulated drugs to the brain, the use of NP to target and destroy tumours or the use of NP as probes for single-molecule tracking in the brain. Brain injection of NP has been used by others for drug delivery purposes^35^,^36^, using local brain-tissue injections. The highly concentrated dose of NP produced by stereotaxic injections presents an obvious risk of inflammation, activating microglia that will engulf large amounts of the injected nano-objects. An alternative to the direct brain injection is to use the blood vascular system, but injected objects can rarely cross a healthy blood–brain barrier. We provide direct evidence of NPs delivered *in vivo* through the brain ventricular system, with no sign of inflammation (activated microglia) and equal dispersion of QD throughout the neocortex, which constitutes a great advantage for single NP tracking. An expected large proportion of injected QD were not co-localized with transfected neurons, even after 1 to 2 h of washing the slices in ACSF, suggesting that the acute slice has a different and more compact extracellular architecture than the organotypic slice in which the same type of QD can be rapidly flushed in and out of the preparation^22^. This is also in accordance to previous studies that showed the reduction of targeting specificity of functionalized NP because of the presence of biological media adsorbed to its surface^37^. The QD-labelled D1R were found moving along the transfected neurons in a similar way as in cultured neurons^9^,^10^. The diffusion coefficients of D1R in spines were smaller than those moving along dendrites, an effect that is likely the sum of two factors: (i) the stabilization of the receptors in the spine head^9^,^10^ and/or (ii) the geometrical effect along the spine neck due to tracking in a very thin tubular shape^38^. In addition, we unveiled that the activation of D1R by its agonist SKF-38393 increased the receptor mobility in acute slice and mobilized a sub-population of fast D1R that may resemble the ‘recycling' ones^28^, a point to be tackled in future investigations. Thus, single neurotransmitter receptor located at the surface of neurons from acute brain slices can be tracked down in living preparations, demonstrating the physiological importance of this process in regulating the dynamic surface distribution and its regulation by the receptor activity status.

It should be noted that our development is currently limited to tagged-receptors since endogenous membrane receptor tracking proved unsatisfactory. Indeed, we performed a series of experiments in which we tracked down an endogenous glutamate receptor (since there is currently no good antibody against extracellular domains of D1R). In naïve brains (no electroporation), the diffusion coefficient distributions from an anti-endogenous receptor or anti-GFP antibodies were similar (Supplementary Fig. 5). This indicates that endogenous and ‘unspecific' antibody/QD complexes provide the same dynamics' signature, precluding any strong conclusion. Furthermore, since most neurotransmitter receptors are widely expressed across the brain and since antibody/QD complexes need to be injected into the lateral ventricle to avoid local inflammation, the penetration of antibodies into the parenchyma will likely crosslink endogenous receptors present at high concentration in the tissue. Thus, the great advantage of anti-tag antibody to freely explore the extracellular environment of the brain, until eventually binding its target on transfected cells, will have to be transposed with innovative engineered strategies to anti-endogenous receptor antibodies.

To conclude, the successful strategy we described could potentially be extended to *in vivo* imaging with the development of new near-infrared nano-probes and imaging techniques, pushing the limits of current high-resolution imaging setups and single-molecule labels. Tracking tagged-receptors provides the advantage to better understand the receptor trafficking using virtually all molecular strategies that modify proteins (for example, mutation, domain deletion and chimaera). Specifically, this new approach will improve our understanding of the mechanisms governing dopamine receptor surface distribution, and shed new lights on the pathological dysregulations affecting these receptors in neuropsychiatric disorders^39^ and neurodegenerative conditions^40^. Since strategies acting on the lateral mobility of D1R and glutamate NMDA receptor in the striatum might improve dyskinesia in L-DOPA-treated animal models of the Parkinson disease^22^, one may envision the surface diffusion of neurotransmitter receptors as a highly valuable therapeutic target. Thus, assessing the mechanisms regulating receptor diffusion and how they could be pathologically affected at the nanoscale may help to provide new strategies to treat neurological and psychiatric disorders.

## Methods

### Animals

Postnatal days 1–4 old Sprague Dawley rats (Janvier, France) were used for this work, both male and female, treated according to the guidelines of the University of Bordeaux/CNRS Animal Care and Use Committee.

### Cell culture and transfection

Cultures of hippocampal neurons were prepared from E18 Sprague Dawley. Briefly, cells were plated at a density of 1.1 × 10^4^ cells cm^−2^ on poly-lysine pre-coated coverslips. Cultures were kept at 37 °C in 5% CO_2_ in a 3% horse serum containing neurobasal medium for the first 3 days after dissection, after which the medium was changed for a serum-free neurobasal medium (Invitrogen). Neurons were co-transfected at 7–10 days *in vitro* with D1-CFP and EGFP using Effectene (QIAGEN) transfection kit. We mixed 2 μg of DNA with 25 μl of Effectene and 8 μl of enhancer in 150 μl of reaction buffer, and then added the mixture to cultured neurons, which were transferred to serum-free neurobasal medium 10 min beforehand. After an incubation period of 45 min, neurons were placed in the old medium again.

### *In utero* electroporation

E16.5 timed-pregnant Sprague Dawley rats were anaesthetized with isofluorane (induction 5% and surgery 2.5%). Animals were kept over a heating blanket (homeothermic monitor Harvard apparatus) to maintain their corporal body at 37 °C, and surgery was done under the light of a cold lamp (Olympus KL1500LCD). Abdominal wall was opened and the uterine horns were carefully exposed and regularly moistened with NaCl 0.9% at 37 °C. DNA solution of pcDNA D1-CFP and pEGFP-N1 (0.5–1 μg μl^−1^ for each plasmid in PBS together with Bromophenol blue) was injected through the uterine wall into one of the lateral ventricle of each embryo (4 μl) with a micropipette (capillaries Harvard apparatus GC100-10 pulled with Nashirige pc-10 and cut to external diameter of 20 μm) linked to a Picospritzer III. Solution rapidly diffuses to the non-injected lateral ventricle. Electroporation was made with forceps-type circular electrodes, 10 mm (platinum Tweezertrode BTX Harvard apparatus), positioned horizontally in order to electroporate cortex and hippocampus. Five electrical pulses (50 V, 50 ms duration and 1 s interval between pulses) were bilaterally delivered with a generator (BTX Harvard apparatus ECM830). The uterine horns were placed back in the abdominal cavity and 15 ml of warm NaCl 0.9% were added. Peritoneal wall and skin were sutured (Ethilon F2413 and Monocryl Y214 respectively) and Buprecare 0.06 μg g^−1^ with Marbocyl 0.016 mg g^−1^ were administered at the end of the surgery.

### Immunostaining of CD11b

Comparison between intra-ventricular and brain-tissue injections of QD were performed in living Sprague Dawley rat pups (1–4 days old). Pups were injected 5 μl in one ventricle and 5 μl in the cortex of the opposite hemisphere, along the same coronal line. Pups were strongly anaesthetized with Isoflurane 3 h after injection and perfused with NaCl 0.9% in water and subsequently with 4% paraformaldehyde (PFA). Brains were then dissected and left in 4% PFA for 24 h. Brains were then left in PBS for 48 h at 4 °C. Coronal slices 50 μm thick were cut in a Vibratome (Leica) and slices close to injection points were recovered and washed thrice with PBS-Triton 0.3%. After incubation with 2% normal donkey serum in PBS-Triton 1.5% to reduce unspecific background, slices were incubated with (1:200) mouse anti-rat integrin alphaM [CD11b] monoclonal antibody (Millipore, #CBL1512Z) overnight at 4 °C. Slices were washed thrice with PBS-Triton 0.3% and finally incubated with secondary antibody donkey anti mouse Alexa 568 (1:1,000) for 4 h at 4 °C. Slices were finally washed thrice with PBS and mounted in glass slices with Vectashield+DAPI (Vector Labs). Images were obtained using an upright fluorescence microscope and the appropriate filters for each staining.

### Single QD tracking in culture

Transfected cultures of hippocampal neurons were mounted in a Ludin chamber (Life Imaging Services) and imaged with an inverted fluorescence microscope. QDs QD655 Goat F(ab')2 anti-Rabbit IgG (Molecular Probes, #Q-11421MP) were first incubated for 30 min with the GFP Rabbit Serum Polyclonal Antibody (Molecular Probes, #A6455). Non-specific binding was blocked by additional casein (Vector Laboratories, USA) to the QD 15 min before use. Neurons were first incubated for 10 min at 37 °C in culture medium with pre-coated QD (final dilution 1:20,000 for anti-GFP coupled QD). Detection of the QD was performed by using a mercury lamp and a filter cube optimized for Qdot 655 Nanocrystals (Semrock Bright-line QD655-C-000). Images were obtained with an integration time of 50 ms acquiring up to 1,000 consecutive frames. Signals were detected using an EMCCD camera (Quantem, Roper Scientific). The effect of SKF-38393 hydrochloride (Sigma-Aldrich, #D047) was tested by directly perfusing a 10 μM solution to the coverslip with either cultured neurons or acute slices for 10 min.

To test whether the proportion of antibodies to QD in the study is adequate for single receptor tracking, a titration of different concentration of antibodies was performed by preparing the QD-anti-GFP complex before incubating with cells at different QD-anti-GFP proportions. Dilutions of QD:anti-GFP ratios of approximately 1:1, 1:10 and 1:1,000 were prepared in culture medium. QD655 Goat F(ab')2 anti-Rabbit IgG were incubated with GFP Rabbit Serum Polyclonal Antibody at the desired ratio and were subsequently incubated with neuronal cultures at DIV 8–10, keeping the QD dilution 1:20,000 in cell media in order to have an equivalent QD number in the three different dilutions. Cells were incubated with the complex for 3 min, and three subsequent washes with medium were performed to remove QD excess. Imaging was performed as described above.

### QD-antibody complexes for *in vivo* labelling of D1 receptors

Following titration experiment results, all experiments in slices were performed at a dilution of approximately 1 QD to 10 anti-GFP. QD-antibody complexes were prepared by incubating 3 μl of QD655 Goat F(ab')2 anti-Rabbit IgG (Molecular Probes, #Q-11421MP) with either 3 μl of GFP Rabbit Serum Polyclonal Antibody (Molecular Probes, #A6455) or 3 μl of FLAG Monoclonal Antibody (Sigma-Aldrich, #2555). The cocktail was then diluted in 54 μl of PBS (final QD concentration of 50 nM), vortexed, briefly spun down and incubated for 30 min at room temperature.

### Intra-ventricular injections

Injections of QD were performed in living Sprague Dawley rat pups (1–4 days old), injecting 5 μl of QD dispersion in each hemisphere at a concentration of 50 nM. Under cold light illumination, the injection place was found by firstly drawing a virtual line between the eye and lambda (easily seen through the skin), secondly finding the midpoint of that line, and thirdly moving 2 mm caudal from that midpoint along the virtual line. The injection was performed in that point, at a depth of 2.6 mm for P1 pups, 2.9 mm for P2 pups, 3.1 mm for P3 pups and 3.5 mm for P4 pups. Injected pups were left back with their mother for 3 h, after which they were killed and brains were dissected. The same procedure was applied for the injection of fluorescently labelled dextrans (Sigma-Aldrich), diluted 1:5 in PBS.

### Acute brain slices preparation

Postnatal day (P) 1–4 Sprague Dawley rats were anaesthetized with isoflurane and parasagittal brain slices (350 μm thick) were prepared in an ice-cold sucrose buffer solution containing (in mM): 250 sucrose, 2 KCl, 7 MgCl_2_, 0.5 CaCl_2_, 1.15 NaH_2_PO_4_, 11 glucose and 26 NaHCO_3_ (gassed with 95% O_2_/5% CO_2_). Slices were then incubated for 30 min at 33 °C and subsequently stored at room temperature in an ACSF solution containing (in mM): 126 NaCl, 3.5 KCl, 2 CaCl_2_, 1.3 MgCl_2_, 1.2 NaH_2_PO_4_, 25 NaHCO_3_ and 12.1 glucose (gassed with 95% O_2_/5% CO_2_; pH 7.35). Slices were used for imaging, 1–4 h after preparation.

### Spinning disk microscopy

QD imaging in acute slices was performed in a Leica DMI6000 inverted microscope (Leica Microsystems) with a Yokogawa spinning disk unit CSU-X1. The setup was equipped with live cell chamber and temperature was constantly kept at 37 °C. Tissue scans were obtained stitching multiple fields of view with either × 10 air, × 63 oil and × 100 oil objectives, with exposure times of 50–100 ms per frame. DAPI staining in fixed tissue was excited with a 405 nm laser line, and detected through a standard DAPI emission filter. EGFP transfections were excited with a 491 nm laser line and emission was observed with a standard GFP filter. For single-QD tracking, QD were excited with a 561 nm diode laser line set at 200 mW, yielding 1.29 mW at the × 63 objective exit, and the emission was filtered with a 650–800 nm band-pass filter. QD tracking images were obtained with × 63 oil objective (Leica HCX PL APO 63 × /1.40-060) and integration times of 20–50 ms acquiring at least 500 consecutive frames for each imaged region. Images were acquired using an Evolve EM-CCD camera (Photometrics), setting the EM gain at 600. The *z* stacks were done with a galvanometric stage (Leica Microsystems) and mosaics were done with a motorized stage Scan IM (Märzhäuser). The system was controlled by MetaMorph software (Molecular Devices).

### Particle tracking

Images were processed with PalmTracer module in Metamorph software (Molecular Devices). The two-dimensional trajectories of single molecules in the plane of focus were constructed by correlation analysis between consecutive images using a Vogel algorithm. The instantaneous diffusion coefficient (D) of QD was calculated for each trajectory, from linear fits of the first four points of the MSD versus time function MSD(t)=4Dt. The pointing accuracy of our system was determined at ∼30 nm.

### Statistical analysis

Comparisons between groups for instantaneous diffusion coefficients were performed using Kolmogorov–Smirnov test (distribution comparison), Wilcoxon matched-pairs signed rank test. Significance levels were defined as **P<0.01.

## Additional information

**How to cite this article:** Varela, J. A. *et al.* Targeting neurotransmitter receptors with nanoparticles *in vivo* allows single molecule tracking in acute brain slices. *Nat. Commun.* 7:10947 doi: 10.1038/ncomms10947 (2016).

## Supplementary Material

Supplementary Information Supplementary Figures 1-5

Supplementary Movie 1 The movie shows an example of QD-anti-GFP complexes (red) in an acute slice with electroporated neurons expressing D1-CFP and cytosolic EGFP (green). Examples of QD moving along the electroporated neuron are indicated with white arrows. Movie was made using MetaMorph (Universal Imaging), Imaris (Bitplane) and Windows Live Movie Maker (Microsoft) software.

Supplementary Movie 2 The movie shows an example of the Multidimensional Image Analysis (MIA) processing of a QD-anti-GFP complex (red) in an acute slice with electroporated neurons expressing D1-CFP and cytosolic EGFP (green). Movie was made using MetaMorph (Universal Imaging), Imaris (Bitplane) and Windows Live Movie Maker (Microsoft) software.

Supplementary Movie 3 The movie shows an example of QD-anti-FLAG complexes (red) in an acute slice with electroporated neurons expressing D1-CFP and cytosolic EGFP (green). Examples of QD moving along the electroporated neuron are indicated with white arrows. Movie was made using MetaMorph (Universal Imaging), Imaris (Bitplane) and Windows Live Movie Maker (Microsoft) software.
