# Supplementary material for: Targeting neurotransmitter receptors with nanoparticles in vivo allows single-molecule tracking in acute brain slices
Source: Nat Commun. 2016 Mar 14;7:10947. doi: 10.1038/ncomms10947 (PMC4793083; doi:10.1038/ncomms10947)
Supplement: Supplementary Information — Supplementary Figures 1-5 [file ncomms10947-s1.pdf]

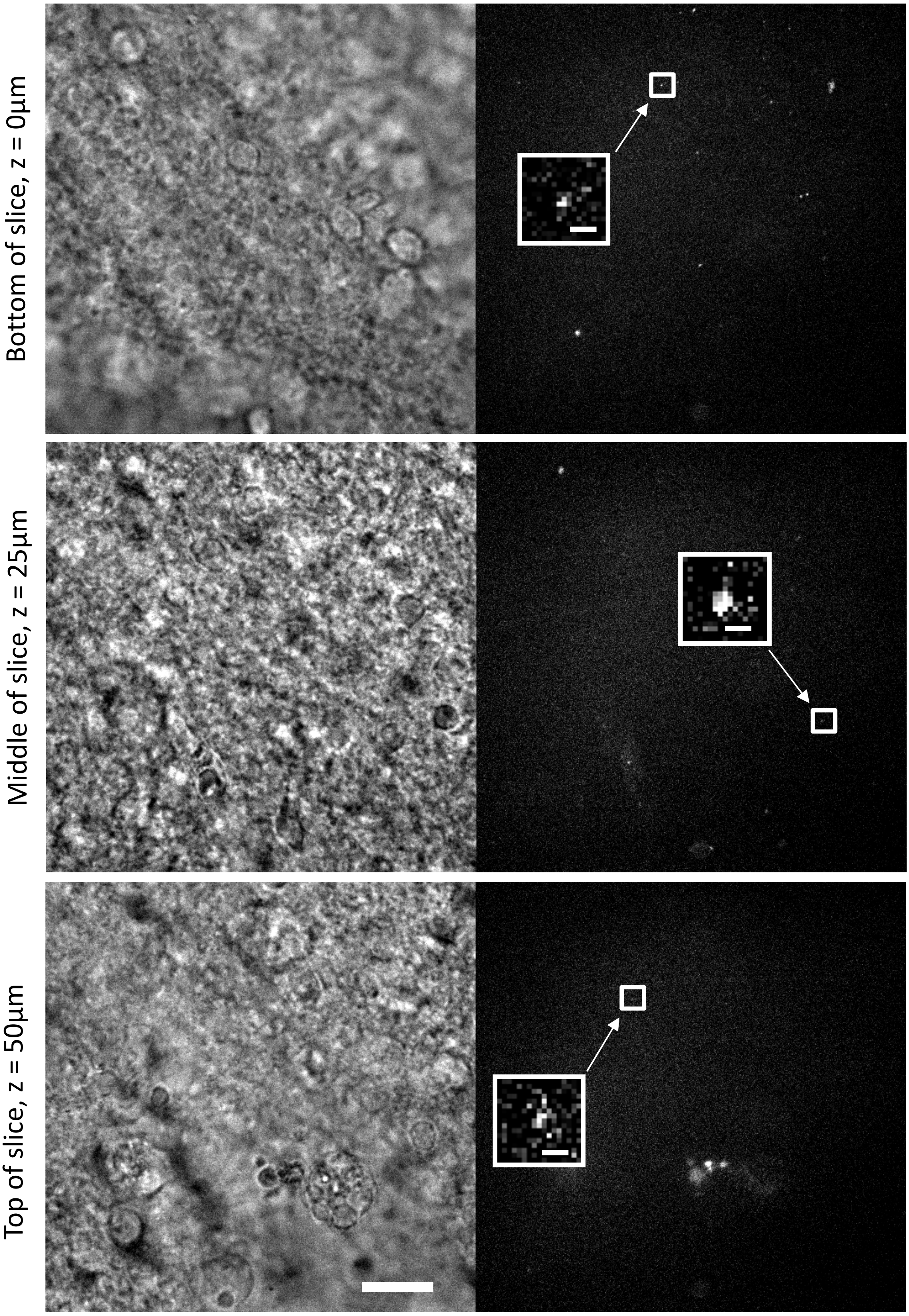

Supplementary Figure 1. Detection of QD in a thin acute slice of 50 µm thickness. The slice can be imaged along the 50 µm in z direction, detecting QD over the entire volume. Examples at the bottom, middle and top of the slice are shown in bright field illumination and QD illumination. Scale bar = 15 µm (main panel), scale bar insets = 600 nm.

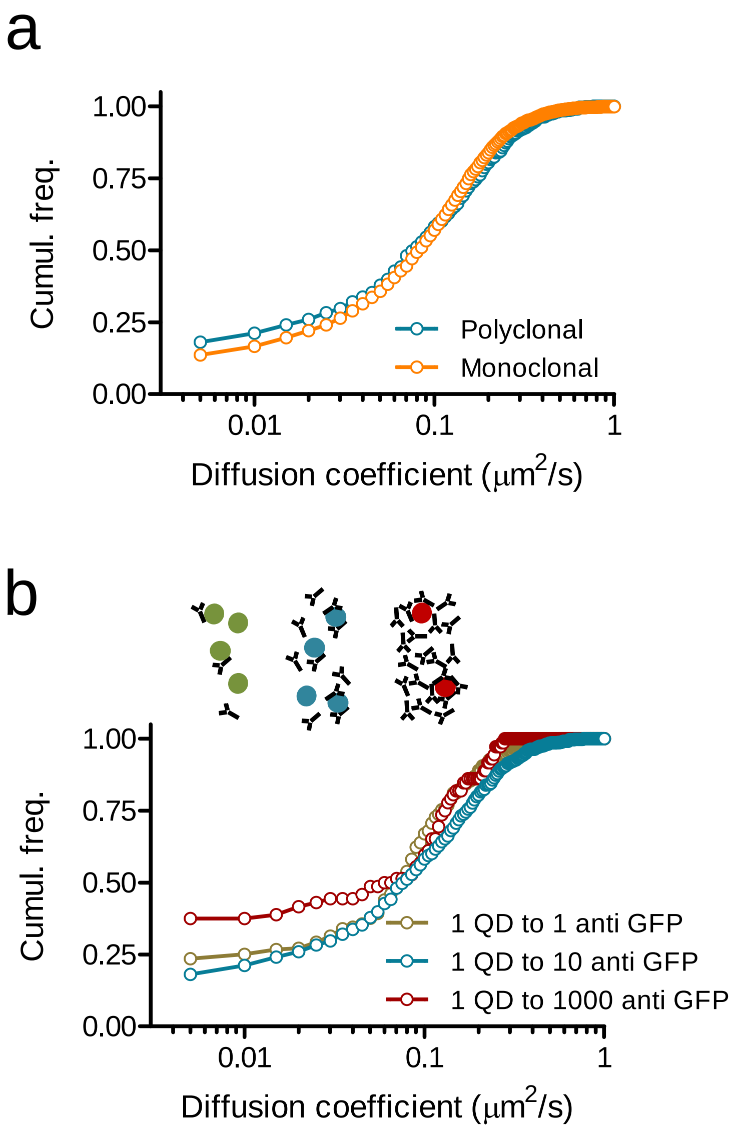

Supplementary Figure 2. (**a**) Comparison between the tracking of single D1R in cultured neurons using polyclonal and monoclonal antibodies (polyclonal antibody 714, n=1205 trajectories; monoclonal antibody, n=3480 trajectories). (**b**) Titration of the proportion between QD and polyclonal antibodies used in cultured neurons to track D1R.

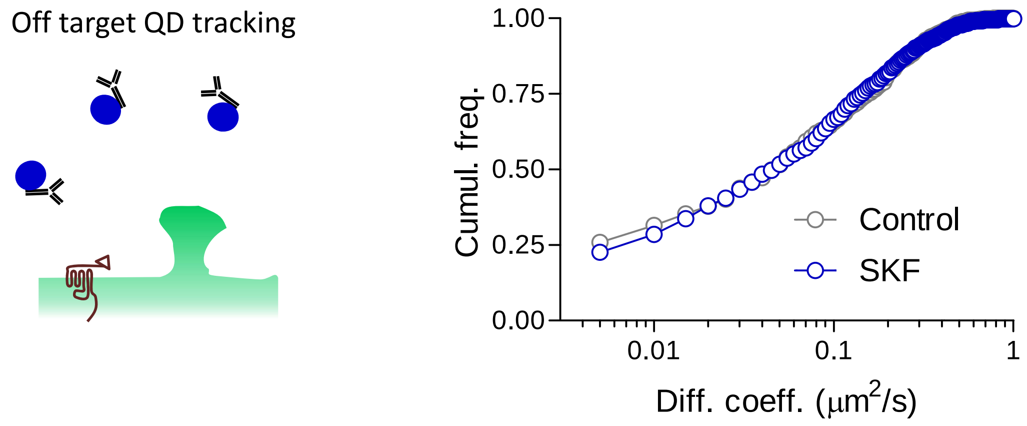

Supplementary Figure 3. The effect of the D1R agonist SKF-38393 (10 μM for 10min) over QD-anti-GFP complexes that are not co-localised with electroporated neurons in acute slices (n=1524 trajectories for Control, n=2260 trajectories for SKF-38393).

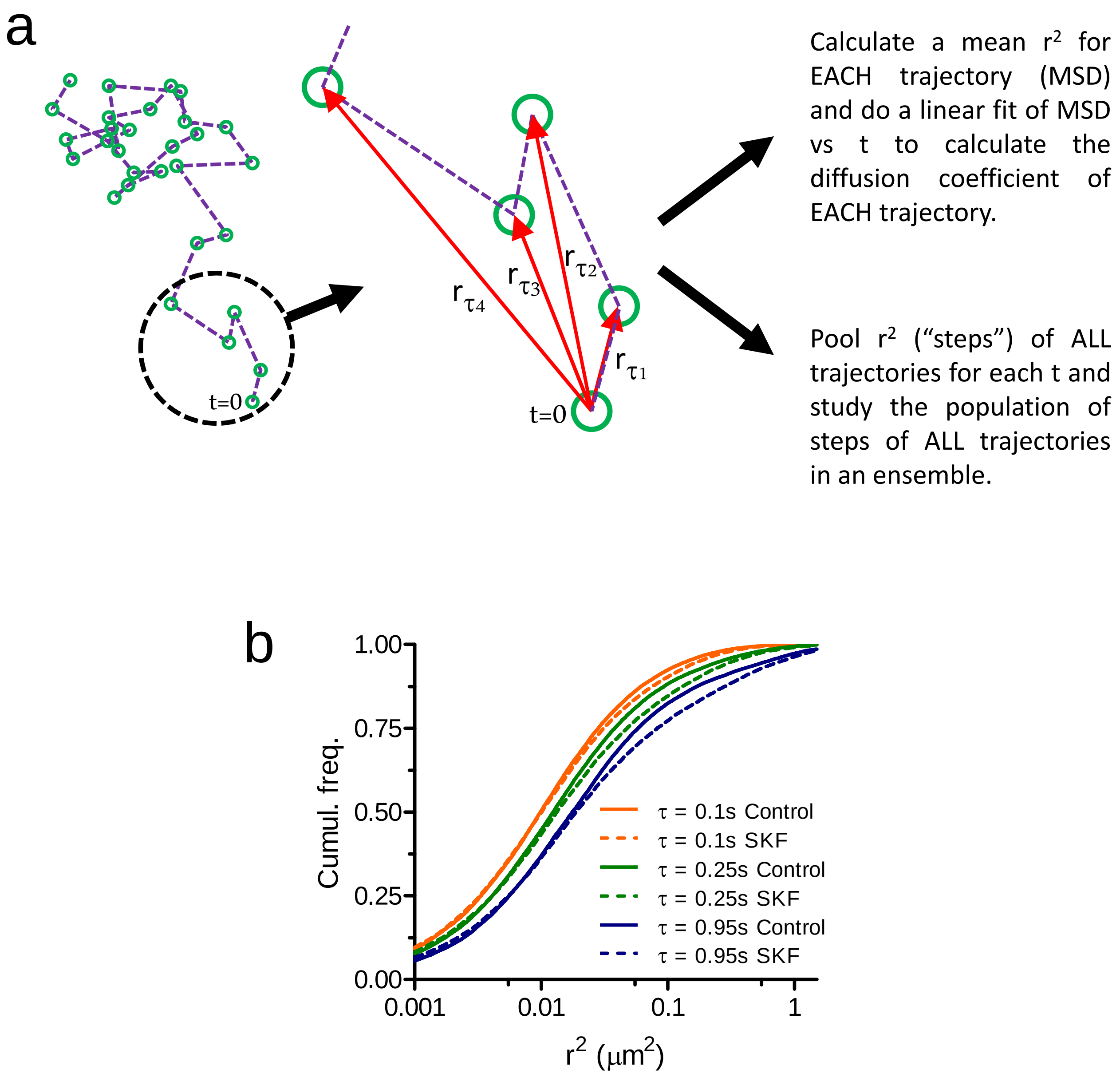

Supplementary Figure 4. (**a**) From each reconstructed trajectory, the displacements between positions at different time lags (t) are used to calculate either the MSD (and subsequently the diffusion coefficient) or the step distribution of the ensemble of trajectories. (**b**) The step size distribution of D1 trajectories in acute slices at different lag times are affected by SKF-38393 treatment, after which steps are shifted to larger displacements.

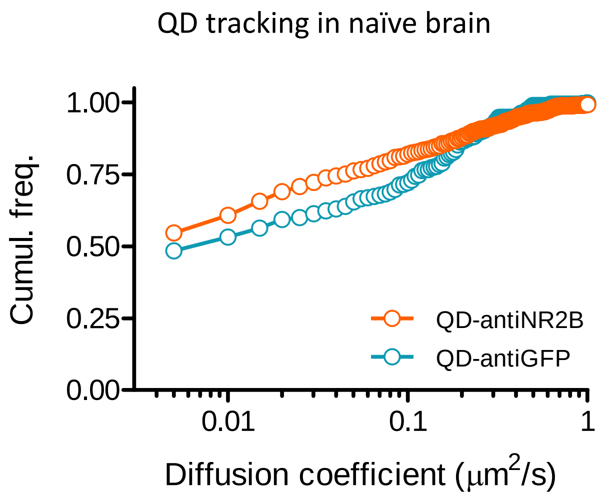

Supplementary Figure 5. Comparison between the tracking of QD-anti-GFP and QD-anti-NR2B in naïve brains (n=446 trajectories for QD-anti-GFP, n=460 trajectories for QD-anti-NR2B).
